# Single‐cell transcriptome characteristics of testicular terminal epithelium lineages during aging in the *Drosophila*

**DOI:** 10.1111/acel.14057

**Published:** 2023-12-03

**Authors:** Xia Chen, Yujuan Qi, Qiuru Huang, Chi Sun, Yanli Zheng, Li Ji, Yi Shi, Xinmeng Cheng, Zhenbei Li, Sen Zheng, Yijuan Cao, Zhifeng Gu, Jun Yu

**Affiliations:** ^1^ Department of Obstetrics and Gynecology, Nantong First People's Hospital Affiliated Hospital 2 of Nantong University; Medical School of Nantong University, Nantong University Nantong Jiangsu China; ^2^ Clinical Center of Reproductive Medicine, Xuzhou Central Hospital Xuzhou Clinical School of Xuzhou Medical University Xuzhou China; ^3^ Institute of Reproductive Medicine Medical School of Nantong University, Nantong University Nantong China; ^4^ Department of Geriatrics Affiliated Hospital of Nantong University, Nantong University Nantong China; ^5^ Department of Rheumatology Affiliated Hospital of Nantong University, Nantong University Nantong China

**Keywords:** aging, *Drosophila*, single‐cell RNA‐sequencing, terminal epithelium, testis

## Abstract

Aging is a complex biological process leading to impaired functions, with a variety of hallmarks. In the testis of *Drosophila*, the terminal epithelium region is involved in spermatid release and maturation, while its functional diversity and regulatory mechanism remain poorly understood. In this study, we performed single‐cell RNA‐sequencing analysis (scRNA‐seq) to characterize the transcriptomes of terminal epithelium in *Drosophila* testes at 2‐, 10 and 40‐Days. Terminal epithelium populations were defined with *Metallothionein A* (*MtnA*) and subdivided into six novel sub‐cell clusters (EP0–EP5), and a series of marker genes were identified based on their expressions. The data revealed the functional characteristics of terminal epithelium populations, such as tight junction, focal adhesion, bacterial invasion, oxidative stress, mitochondrial function, proteasome, apoptosis and metabolism. Interestingly, we also found that disrupting genes for several relevant pathways in terminal epithelium led to male fertility disorders. Moreover, we also discovered a series of age‐biased genes and pseudotime trajectory mediated state‐biased genes during terminal epithelium aging. Differentially expressed genes during terminal epithelium aging were mainly participated in the regulation of several common signatures, e.g. mitochondria‐related events, protein synthesis and degradation, and metabolic processes. We further explored the *Drosophila* divergence and selection in the functional constraints of age‐biased genes during aging, revealing that age‐biased genes in epithelial cells of 2 Days group evolved rapidly and were endowed with greater evolutionary advantages. scRNA‐seq analysis revealed the diversity of testicular terminal epithelium populations, providing a gene target resource for further systematic research of their functions during aging.

## Graphical abstract

Transcriptome characteristics of testicular terminal epithelium populations.

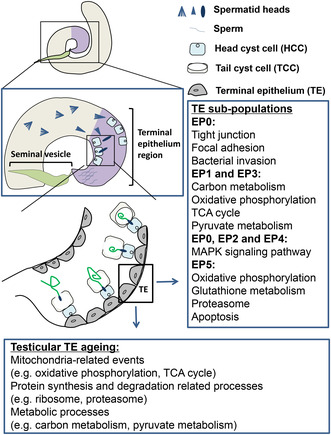

**Abbreviations**
Act5C: Actin 5C
ANOVA: analysis of variance
Atg8b: Autophagy‐related 8b
BSA: bovine serum albumin
CadN: Cadherin‐N
CySCs: somatic cyst stem cells
DEGs: differentially expressed genes
Dp/E2f1: DP transcription factor/E2F transcription factor 1
Shg: E‑cadherin
ECM: extracellular matrix
Fas3: Fasciclin III
FISH: Fluorescent In Situ Hybridization
GO: Gene Ontology
GBs: generate gonialblasts
GSCs: germline stem cells
GstD1: glutathione S transferase D
HBSS: Hanks Balanced Salt Solution
hh: hedgehog
KEGG: Kyoto Encyclopedia of Genes and Genomes
lncRNAs: long non‐coding RNA
MTF‐1: Metal response element‐binding Transcription Factor‐1
MtnA: Metallothionein A
MAPK: mitogen activated protein kinase
Mst84Db: Male‐specific RNA 84Db
Mst87F: Male‐specific RNA 87F
mt:ATPase6: mitochondrial ATPase subunit 6
Ndae1: Na+‐driven anion exchanger 1
PBST: PBS‐Triton X‐100
PBS: phosphate‐buffered saline
qRT‐PCR: quantitative real‐time reverse transcription PCR
dN/dS: ratio of non‑synonymous to synonymous substitutions
Scpr‐A: SCP‐containing protein A
scRNA‐seq: single‐cell RNA‐sequencing
SV: seminal vesicle
snRNA‐seq: single‐nucleus RNA‐sequencing
Ssk: Snakeskin
t‐SNE: t‐distributed Stochastic Neighbor Embedding
TE: terminal epithelium
Tsp2A: Tetraspanin 2A
Tsf2: Transferrin 2
TCA: tricarboxylic acid
UMIs: unique molecular identifiers
Wnt6: Wnt oncogene analog 6

## 1 INTRODUCTION

Aging is characterized by a progressive loss of physiological integrity, manifesting as cellular senescence, genomic instability, degraded telomeres, epigenetic changes, exhausted stem cells, mitochondrial dysfunction, protein homeostasis loss, dysregulated sensing of nutrients, and intercellular communication changes (López‐Otín et al., 2013). Reproductive organs and tissues suffer morphological and functional changes during aging, which gradually lead to decreased male fertility (Zhuang et al., 2013). *Drosophila* epithelial cells comprise the framework structures that maintain the stable existence of testicular tissue. However, there has been little research on the aging process of testicular epithelial cells. Long term observations in *Drosophila* showed that testicular morphology becomes thinner during aging. Moreover, it was speculated that epithelial cells play hitherto underestimated roles during aging of the testis.

Among many species, the well understood process of spermatogenesis shows distinct conservation (Barreau et al., 2008; White‐Cooper, 2010). Two stem cell lineages, somatic cyst stem cells (CySCs) and germline stem cells (GSCs), ultimately produce sperm and their differentiation and self‐renewal are maintained by hub cells originating from somatic epithelial cells (Carbonell et al., 2017; Chang et al., 2019). Mature cyst cells differentiate from CySCs, providing an environment conducive to encapsulating somatic cyst cells, thus inducing the growth and differentiation of germ cells (Amoyel et al., 2016). In the testicular niche, GSCs divide asymmetrically to generate gonialblasts (GBs) and then experience 4 cycles of mitosis, followed by meiosis, to produce spermatocytes and spermatids and then mature sperm (Demarco et al., 2014). During the final stage of spermatogenesis, an epithelial region, called the terminal epithelium (TE) region, anchors the head end of bundles of elongated spermatids during individualization and coiling at the tail of the testis, helping to release sperm into the seminal vesicle (SV) (Dubey et al., 2019). However, the regulatory functions and the transcription signatures of the TE region are unknown.

Single‐cell transcriptome sequencing (scRNA‐seq) technology is used for the high‐throughput detection of transcription signatures at single‐cell resolution, and is widely used in studies of the reproductive system (Shi et al., 2021; Tan et al., 2023; Zhao et al., 2020). Recently, several scRNA‐seq studies were performed to generate a single‐cell atlas or cellular landscapes in different study models in *Drosophila* testis (Witt et al., 2019; Yu, Fu, et al., 2023; Yu, Li, et al., 2023). All these scRNA‐seq studies identified epithelial cell populations with under‐appreciated functions in testicular biology. By using droplet‐based 10x Genomics and plate‐based Smart‐seq2 sequencing strategies, a unified single‐nucleus RNA‐sequencing (snRNA‐seq) on isolated cell nuclei is performed for the entire adult *Drosophila*, providing a significant resource platform in the FlyCell Atlas database (https://flycellatlas.org/) (Li et al., 2022). Analysis of snRNA‐seq data at the FlyCell Atlas and functional experimental verification using In Situ Hybridization revealed that *Metallothionein A* (*MtnA*) was the most highly upregulated and enriched gene in the TE region of *Drosophila* testis (Raz et al., 2023; Zhao et al., 2010).

Herein, scRNA‐seq analysis was carried out in *Drosophila* testis, and the characteristics and transcription signatures of TE populations during aging in *Drosophila* were investigated. TE populations were identified by their expression of *MtnA*, and the characteristics and their transcription signatures were inferred. The results provide a comprehensive understanding of TE populations and highlights that the single‐cell‐based strategy offers novel targets for TE‐mediated spermatid release and maturity during aging.

## 2 RESULTS

### 2.1 scRNA‐seq of *Drosophila* testicular epithelial cells in testis

**FIGURE 1:**
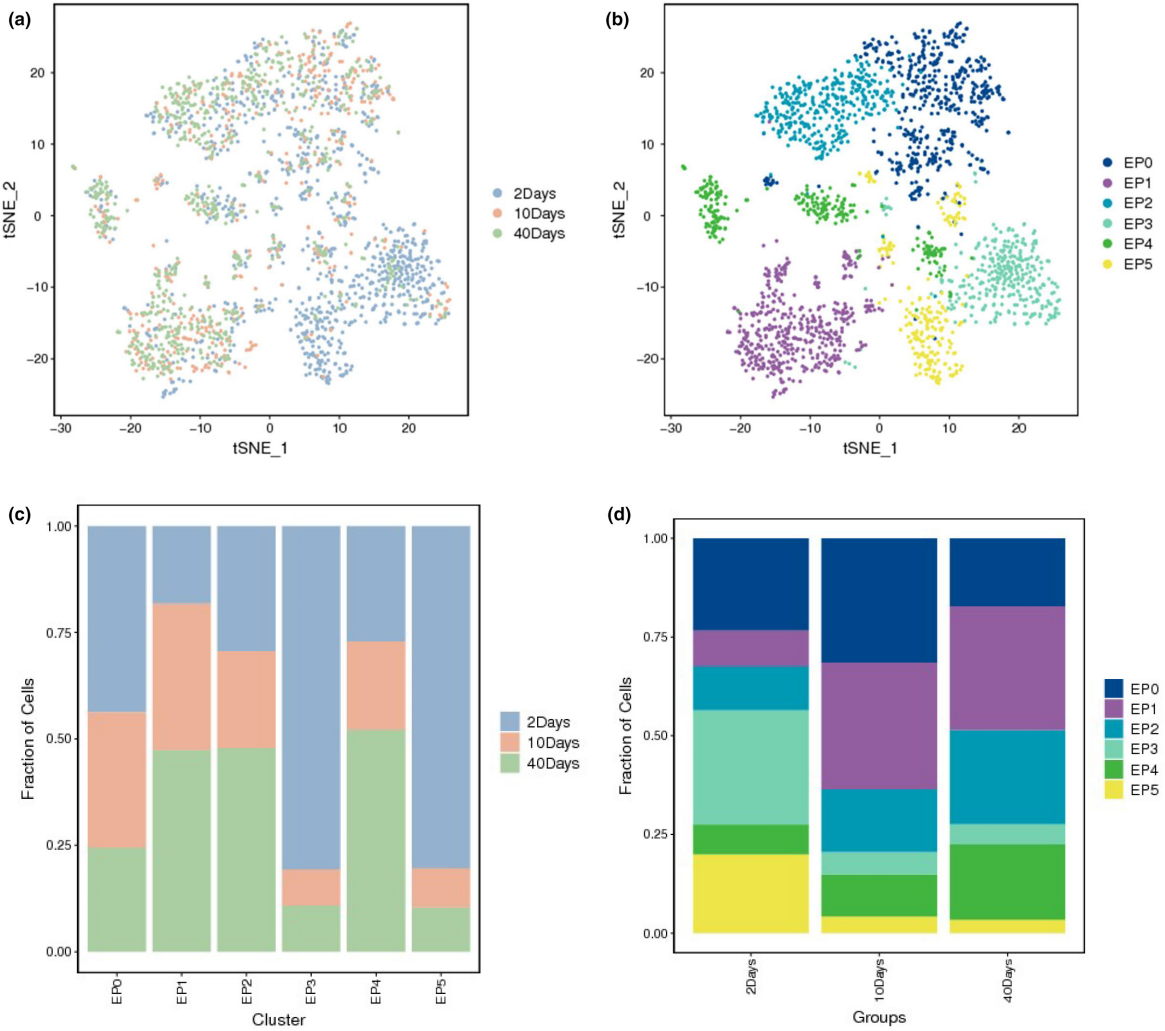
Identification of testicular TE populations using scRNA‐seq. (a) tSNE visualization of testicular TE populations during aging. (b) tSNE visualization of sub‐cell clusters of TE populations. (c) Cellular components of different time groups for each sub‐cell cluster of testicular TE populations. (d) Cellular components of sub‐cell clusters of testicular TE populations at 2, 10 and 40 days.

scRNA‐seq of *Drosophila* testis was carried at 2, 10 and 40 days to assess epithelial cell populations and their heterogeneity during aging. After filtering, 2114 high quality epithelial cells were retained, with a median of 1215.5 genes and 6099 unique unique molecular identifiers (UMIs) per cell. According to recently reported studies and our previously established marker genes (Witt et al., 2019; Yu, Fu, et al., 2023; Yu, Li, et al., 2023), we identified the testicular TE populations using *MtnA* gene expression. Testicular TE populations were further subdivided into six novel cell clusters (EP0–EP5). The distributions of TE populations at different time points or sub‐cell populations were merged using tSNE (Figure 1a,b).

Next, nUMIs and nGenes were used to quantify the RNA contents and gene expressions in the testicular TE sub‐cell clusters (Figure S1a,b), which showed that the EP3 and EP5 populations had relatively lower numbers of nUMIs than the other testicular TE populations. We also noticed that both the nUMIs and nGenes of all sub‐cell clusters increased dramatically during testicular TE aging (Figure S1c,d and Tables S1 and S2). Overall, during testicular TE aging, the fraction of EP0, EP3 and EP5 cell populations were dramatically reduced, while those of the EP1, EP2 and EP4 cell populations were obviously increased (Figure 1c,d). Using these data, we roughly identified two types of epithelial population trends during testicular TE aging.

### 2.2 Diversity of testicular TE populations and their characteristics

**FIGURE 2:**
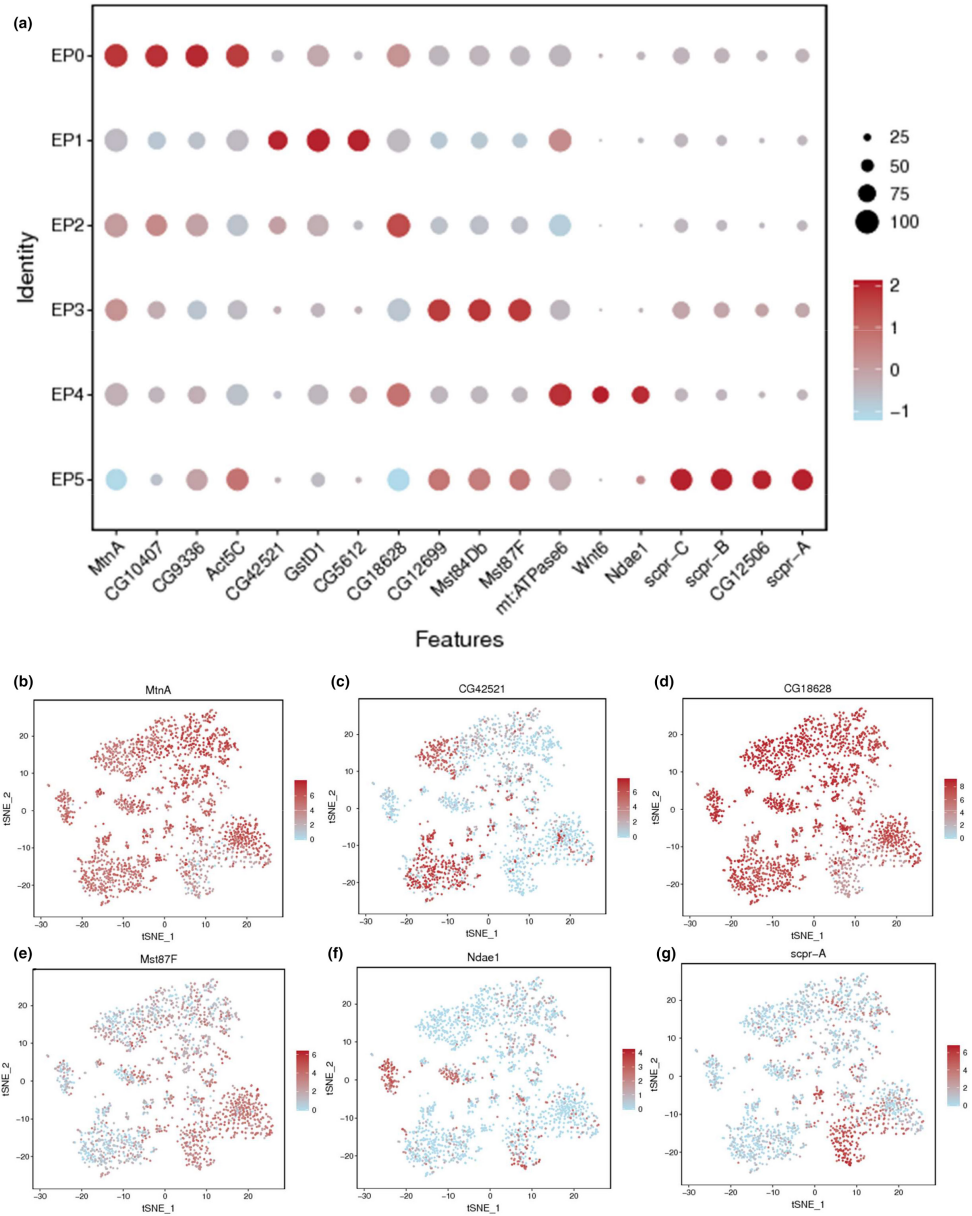
Annotation of testicular TE populations at single cell resolution. (a) Dotplot view of representative marker genes. The dot's diameter is related to the proportion of cells expressing each gene in each sub‐cell cluster. The intensity of the color represents the average normalized expression level. (b–g) tSNE visualizations of testicular TE populations for *MtnA* (b), *CG42521* (c), *CG18628* (d), *Mst87F* (e), *Ndae1* (f) and *Scpr‐A* (g).

To date, sub‐classes within TE cells have not been described in *Drosophila* testis. To explore the characteristics of the testicular TE populations, we annotated the cell population subclasses as EP0–EP5 and identified a series of abundantly expressed marker genes in the testicular TE sub‐cell types. Figure 2 and Table S3 show the Dotplot and tSNE visualization of key marker genes.

The majority of testicular TE populations expressed *MtnA*, which was also identified as a representative marker gene for EP0 (Figure 2a,b). Other representative marker genes, such as *CG10407*, *CG9336*, and *Act5C* (encoding Actin 5C), were enriched in the EP0 population (Figure 2a). We determined EP1 populations based on the expression of *CG42521*, *GstD1* (encoding glutathione S transferase D), and *CG5612* (Figure 2a,c). Moreover, *CG18628* was identified as a representative marker gene for the EP2 population (Figure 2a,d). The EP3 population showed high expression of *CG12699*, *Mst84Db* (encoding Male‐specific RNA 84Db), and *Mst87F* (encoding Male‐specific RNA 87F) (Figure 2a,e). The EP4 population showed high expression of *mt:ATPase6* (encoding mitochondrial ATPase subunit 6), *Wnt6* (encoding Wnt oncogene analog 6), and *Ndae1* (encoding Na + −driven anion exchanger 1) (Figure 2a,f). Furthermore, our scRNA‐seq analysis identified that the EP5 population (Figure 2a,g) expressed high levels of *CG12506* and *Scpr‐A* (encoding SCP‐containing protein A), *Scpr‐B*, and *Scpr‐C*.

To further underline the transcription networks of testicular TE populations, we performed GO and KEGG enrichment analysis for marker gene collections in the sub‐class populations. Top 20 enriched KEGG pathways showed that the EP0 population was related to tight junctions, focal adhesion, and bacterial invasion of epithelial cells, and various signaling pathways, such as the Ras signaling pathway, the Rap1 signaling pathway, and the Toll and Imd signaling pathway (Figure S2a). Interestingly, the EP0, EP2, and EP4 populations showed similar signature cross‐talks for abundantly expressed genes (Figure S2a–c). For instance, we noticed all three TE populations were related to the mitogen activated protein kinase (MAPK) signaling pathway. Moreover, the EP1 and EP3 populations were predicted to participate in the regulation of multiple metabolic pathways, such as carbon metabolism, oxidative phosphorylation, the tricarboxylic acid (TCA) cycle, and pyruvate metabolism (Figure S2d,e). The EP5 population exhibited some similar characteristics to EP1 and EP3, and was predicted to be involved in oxidative phosphorylation, glutathione metabolism, proteasome, and apoptosis (Figure S2f).

To deepen the understanding of relationships between TE transcription features and testicular functions, we knocked down three candidate genes that were involved in the regulation of enriched pathways. The Esg‐GFP was found with expression in TE populations in the *esg > GFP* testis (Figure S3a). In particular, knockdown of *blw*, *PCB* and *Fum3* genes driven by esg‐Gal4 led to severe male reproductive disorders (Male fertility rate: *esg>*, 100%, *n* = 20; *esg > blw RNAi*, 4.8%, *n* = 21; *esg > PCB RNAi*, 16%, *n* = 25; and *esg > Fum3 RNAi*, 0%, *n* = 18). Among them, we found that knockdown of these genes featured massively enlarged TE regions that might impair male fertility (Figure S3b). Moreover, FasIII signals were reduced in TE regions after disturbing TE relevant pathways, demonstrating that tight junctions and adhesion were important for TE populations in *Drosophila* testes (Figure S3c). Together with these data, we have revealed that the diversity of TE cell populations may have significant impacts on the reproductive outcomes in testes.

### 2.3 Representative marker genes for testicular TE aging

**FIGURE 3:**
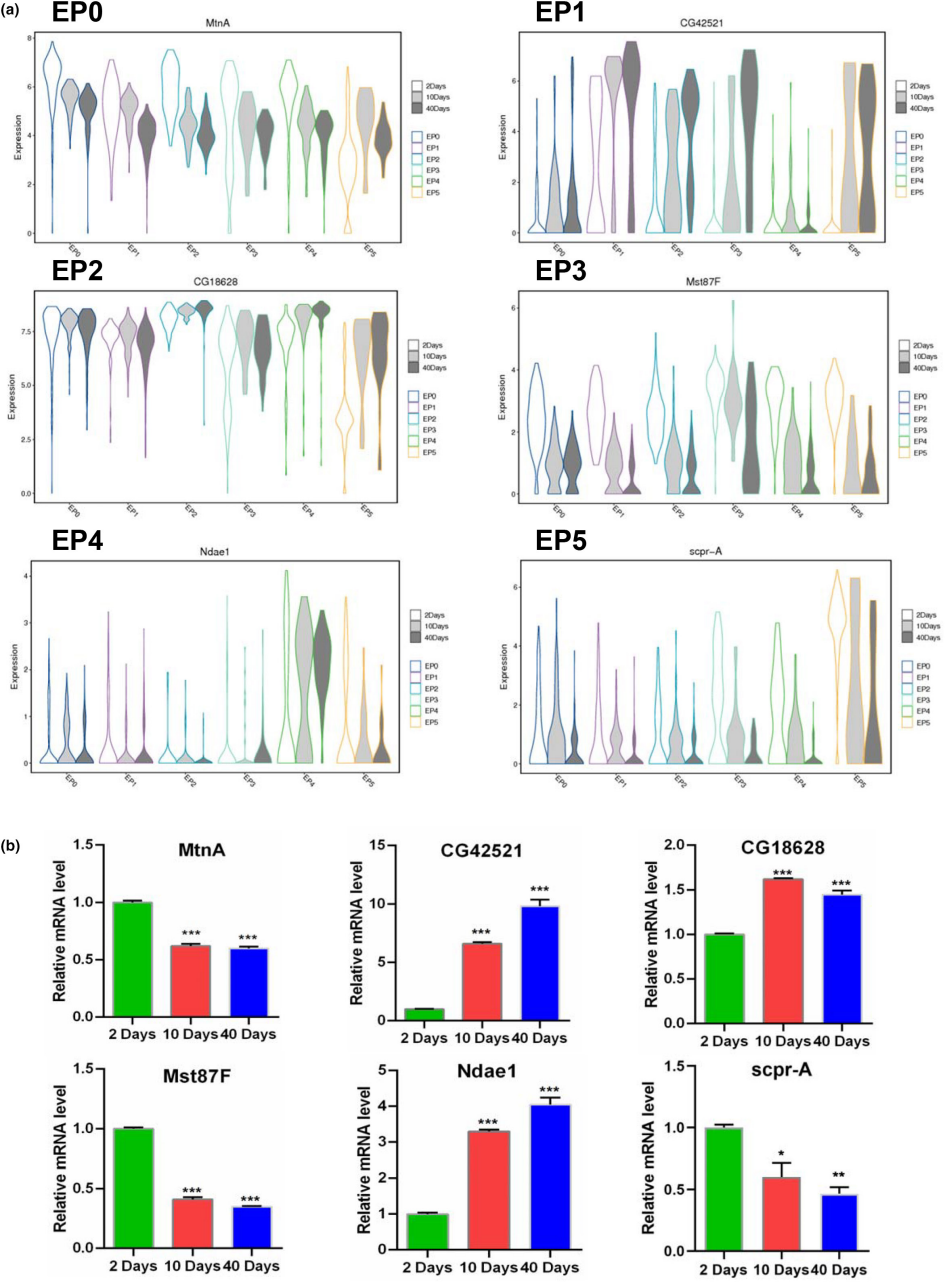
Expression pattern analysis of representative marker genes during aging. (a) Violin plots of *MtnA*, *CG42521*, *CG18628*, *Mst87F*, *Ndae1* and *Scpr‐A* for each sub‐cluster of testicular TE populations during aging. (b) Relative mRNA levels of *MtnA*, *CG42521*, *CG18628*, *Mst87F*, *Ndae1*, and *Scpr‐A* in *Drosophila* testes. *p < 0.05, ***p < 0.001.

To further understand the characteristics of sub‐cell populations during testicular TE aging, we investigated representative marker gene expression patterns at 2, 10 and 40 Days. Violin plots of representative marker genes were constructed for sub‐cell clusters of testicular TE populations in the three age groups (Figure 3a and Figure S4a–d). The expression of *MtnA* (an EP0 marker gene) was significantly downregulated during aging in EP0‐EP4 populations, but not in the EP5 population. *CG42521* (an EP1 marker gene) expression was dramatically upregulated, while *Mst87F* and *CG12699* (EP3 marker genes) expression levels were obviously downregulated in the testicular TE populations during aging. Moreover, *CG18628* (an EP2 marker gene) expression was upregulated in the vast majority of sub‐cell clusters of testicular TE populations, while *Ndae1* (an EP4 marker gene) expression was obviously upregulated in the EP4 population during aging. We also noticed that the expression levels of multiple representative EP5 marker genes (*Scpr‐A*, *Scpr‐B*, *Scpr‐C*, and *CG12506*) dramatically decrease in the testicular TE populations during aging. Next, we assessed the relative mRNA levels of representative marker genes for testicular TE in the *Drosophila* testis using qRT‐PCR, which revealed similar expression patterns during testicular TE aging (Figure 3b and Figure S4e–h). We further verified localizations and expression patterns of *MtnA*, *CG42521*, *CG18628*, *Mst87F*, *Ndae1*, and *Scpr‐A* via Fluorescent In Situ Hybridization (FISH) assays in testicular TE at 2 Days and 40 Days. These marker genes were all detected in TE cells and their expression trends were consistent with scRNA‐seq data (Figure S5). The above data preliminarily clarified the characteristics of representative marker genes during testicular TE aging.

### 2.4 Trends and differentially expressed genes (DEGs) analysis of age‐biased genes during testicular TE aging

**FIGURE 4:**
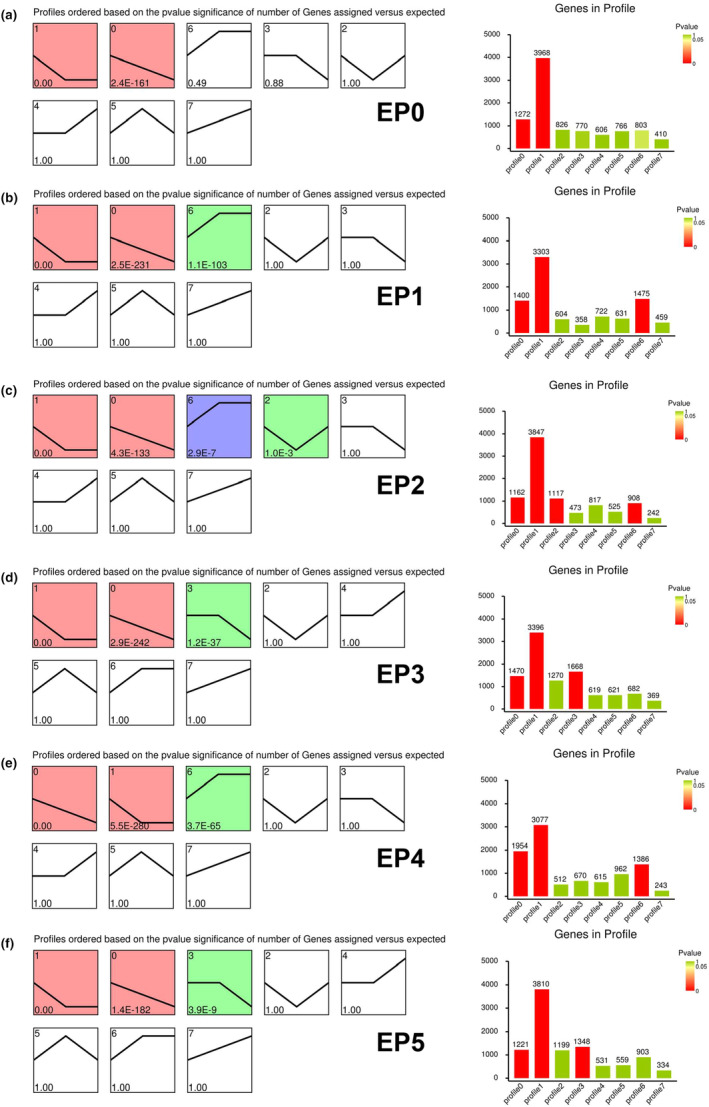
Trends in the expression levels of genes in testicular TE populations. Gene expression trend analysis of EP0 (a), EP1 (b), EP2 (c), EP3 (d), EP4 (e) and EP5 (f) TE populations and the number of genes identified in each trend profile.

To identify age‐biased genes, we next explored the expression trends during testicular TE aging. Interestingly, we discovered a series of age‐biased genes with certain expression trends in various testicular TE populations (Figure 4). We next pinpointed the relevant trends of representative marker genes for testicular TE, calculated using their average expression level values at 2, 10 and 40 Days. Specifically, several marker genes, such as *MtnA*, *CG42521*, *Mst87F*, and *Scpr‐A*, showed similar expression trends in corresponding sub‐cell clusters of testicular TE populations during aging (Figure S6).

To investigate the transcription regulation of testicular TE aging at single‐cell resolution, we analyzed DEGs between 2 Days and 40 Days groups, and their enrichment from each sub‐cell clusters. Interestingly, KEGG pathway analysis revealed several common signatures that were highly enriched in most of the TE populations during aging of the *Drosophila* testis (Figure S7). These signatures comprised mitochondria‐related events (e.g. oxidative phosphorylation, TCA cycle), protein synthesis and degradation‐related processes (e.g. ribosome, proteasome), and metabolic processes (e.g. carbon metabolism, pyruvate metabolism). Taken together, these data provided clues to the characteristics of molecular regulation during testicular TE aging.

### 2.5 Three novel clusters for testicular TE aging

To further investigate the testicular TE features, pseudotime trajectory analysis of testicular TE populations during aging was carried out, which identified three TE clusters in *Drosophila* testis (Figure S8a–c). Analysis of each cluster's cellular components showed that the fraction of testicular TE populations conformed to the pseudotime trajectory (State 1 to State 3) direction from EP5 to EP0 (Figure S8d,e; Figure S9a). Moreover, we also observed that testicular TE populations in State 1 mainly comprised epithelial cells in 2 days group, while testicular TE populations in State 2 and State 3 were likely to be clustered with epithelial cells in 10 days and 40 days groups (Figure S8f,g and Figure S9b,c).

**FIGURE 5:**
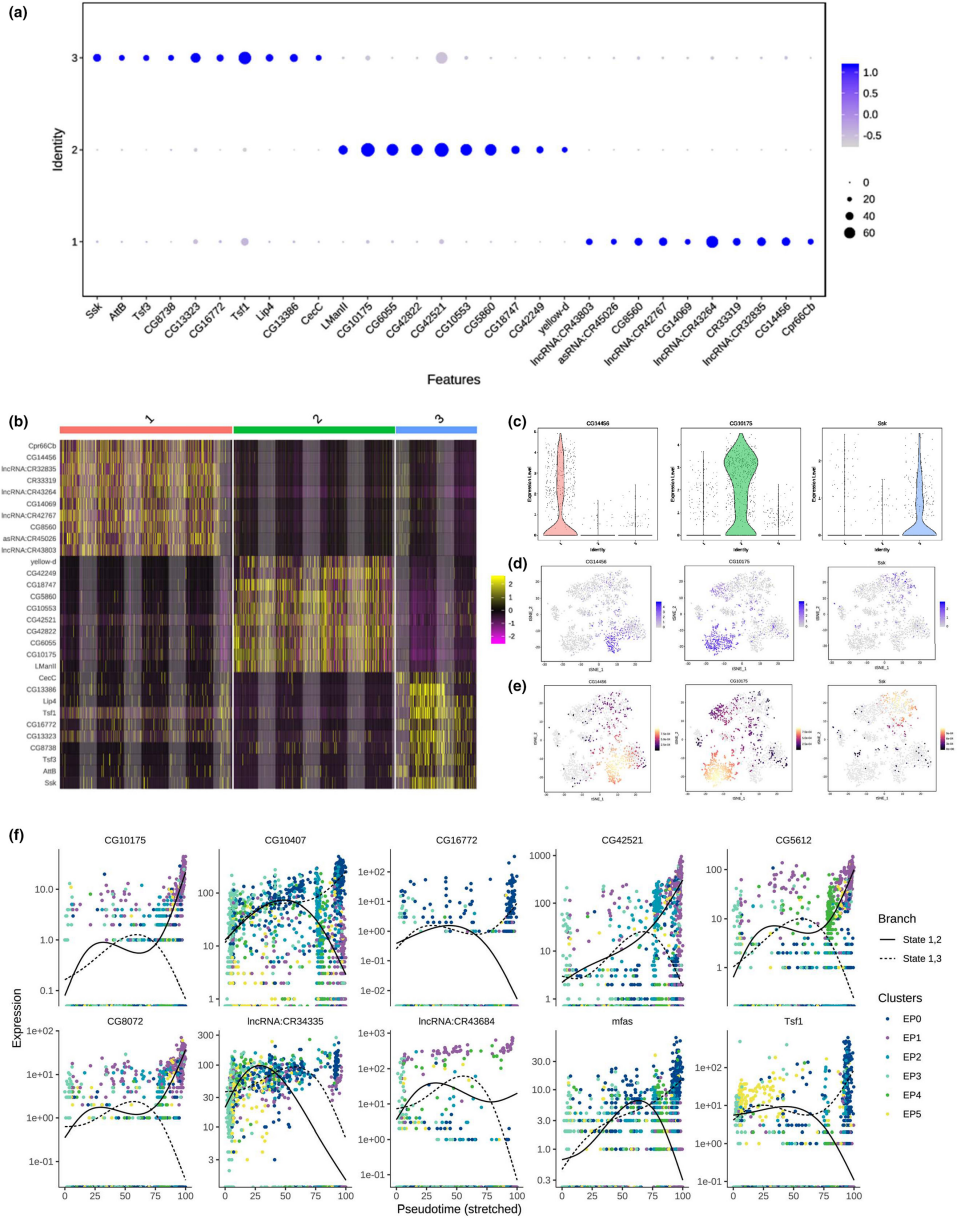
Characteristics of the three clusters of testicular TE populations. (a) Dotplot of representative highly expressed genes in each cluster. (b) Heatmap view of representative highly expressed genes in each cluster. (c) Violin plot views of *CG14456*, *CG10175*, and *Ssk* expression levels in each cluster. (d) tSNE visualizations of *CG14456*, *CG10175*, and *Ssk* expression levels in testicular TE populations. (e) Density plot views of *CG14456*, *CG10175*, and *Ssk* expression levels in testicular TE populations. (f) Spline plots of ten representative state‐biased genes in testicular TE populations.

Next, we analyzed the state‐biased genes in testicular TE populations, which were visualized using dot plots and heatmap views of the genes with the highest expression in a state‐biased manner (Figure 5a,b). Violin plot views of *CG14456*, *CG10175*, and *Ssk* (encoding Snakeskin) were shown for representative state‐biased genes in State 1, State 2 and State 3, respectively (Figure 5c). Meanwhile, tSNE visualizations and the density plots of *CG14456*, *CG10175*, and *Ssk* in each stage showed the same results (Figure 5d,e). Consistent with the pseudotime trajectory analysis in testicular TE populations, we also identified many genes that showed specific state‐biased expression based on their expression patterns of various cell populations. We listed the ten representative state‐biased genes that were responsible for the cluster‐mediated branches, and used spline plots to reveal the expression dynamics of the two branches (Figure 5f).

Then, DEGs with dynamic changes in their gene expression patterns in each testicular epithelial state between the 2 days and 40 days groups were analyzed. The expression patterns of six downregulated marker genes that were identified in all three clusters of TE populations were shown in a heatmap (Figure S10a). Unexpectedly, we discovered that ribosomal RNA and long non‐coding RNA (lncRNAs) might play key roles during testicular TE aging. Among them, *lncRNA:CR34335*, *lncRNA:CR43684*, and *lncRNA:Hsromega* were representative, significantly enriched lncRNAs, which according to tSNE map visualization, were markedly downregulated during testicular TE aging (Figure S10b–d). We also performed KEGG analysis of each cluster for DEGs between the 2 days and 40 days groups. Notably, protein synthesis‐related processes (e.g. ribosomes) were markedly enriched in all clusters during testicular TE aging (Figure S10e–g). However, many different signatures also existed in each cluster. In addition to events related to protein synthesis, protein degradation‐related processes (e.g. proteasome) were also active in cluster 1 of testicular TE populations during aging (Figure S10e). The TCA cycle and multiple metabolic processes, such as pyruvate metabolism, carbon metabolism, glycolysis/gluconeogenesis, and the biosynthesis of amino acids, were highly enriched in cluster 2 of testicular epithelial populations during aging (Figure S10f). Moreover, multiple signatures, including the Estrogen signaling pathway and the Toll and Imd signaling pathway, appear to be active in cluster 3 of testicular epithelial populations during aging (Figure. S10g). Taken together, these data provided key resources and novel targets for testicular TE aging in *Drosophila*.

### 2.6 dN/dS trends of age‐biased genes during testicular TE aging

**FIGURE 6:**
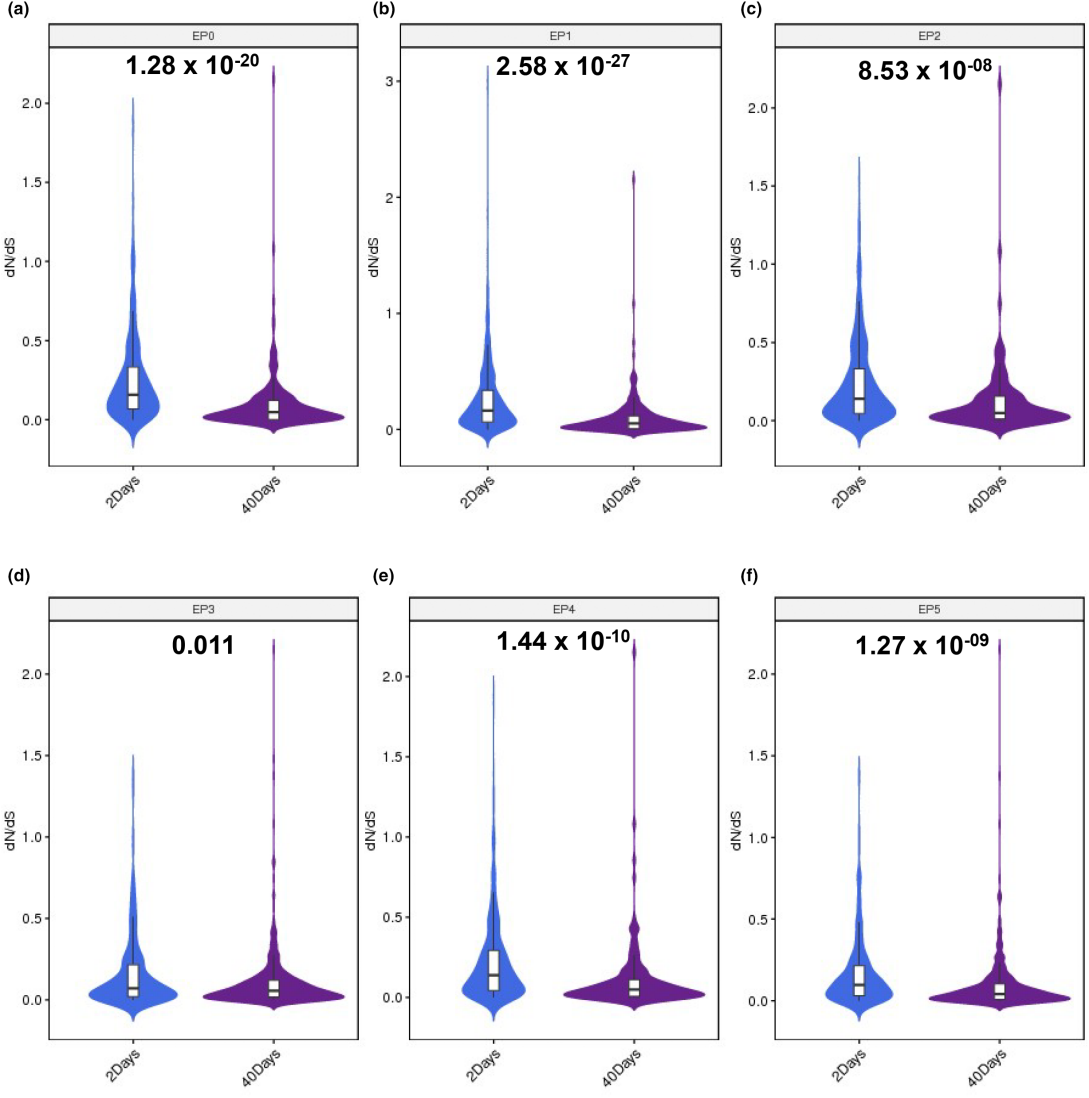
dN/dS trends of TE populations during aging. Age‐biased genes in EP0 (a), EP1 (b), EP2 (c), EP3 (d), EP4 (e) and EP5 (f) TE populations between the 2 days group and the 40 days groups. dN/dS values are from flyDIVaS. Genes with a dS value = 0 (or no numerical value, or both dN and dS value = 0, or an abnormal dN/dS value) were deleted.

Based on data from flyDIVaS30 (Stanley Jr. & Kulathinal, 2016), a genomics resource for comparing *Drosophila* divergence and selection, we compared the ratio of non‐synonymous to synonymous substitutions (dN/dS) values for age‐biased genes in each sub‐cell cluster of epithelial populations in the *Drosophila* testis. Surprisingly, we found that in all the sub‐cell clusters of testicular TE populations, genes biased in the TE cells of the 2 days group had higher dN/dS compared with the genes biased in TE cells of the 40 days group (Figure 6).

## 3 DISCUSSION

In the *Drosophila* testis, the spermatid cyst enters the TE region, and during spermatid release, spermatids are pulled back from the head cyst cells with their tails leading through the testicular duct (Dubey et al., 2016). To date, the reproductive function of TE region has barely been explored, and it was even unclear whether TE populations are important for the testis. Recently, Autophagy‐related 8b (Atg8b), identified as a ubiquitin‐like protein, was observed to be expressed in a male germline specific manner and is required for male fertility, independent of lipidation and autophagy (Jipa et al., 2021). Interestingly, Atg8b mutation caused a massively enlarged TE region and the accumulation of immobile sperm (Jipa et al., 2021), suggesting key roles for TE populations in spermatid release and maturation. However, the regulatory mechanism of TE region remains largely unknown.

Aging is characterized by various features, such as gradually decreasing stem cell functionality, leading to reduced tissue regenerative potential (Rando, 2006). Age‐associated changes in the stem cell niches directly influence stem cell function and self‐renewal ability, and are observed in many tissues (Wallenfang, 2007; Boyle et al., 2007; Chakkalakal et al., 2012). A previous study demonstrated that Lin28 inhibition decreased *upd* expression levels, leading to hub cell aging and loss of the stem cell niche in old testis (Sreejith et al., 2019). MicroRNA miR‐9a is upregulated in male aging GSCs, in which it targets and downregulates the adhesion protein Cadherin‐N (CadN) to enable GSC detachment toward terminal differentiation during spermatogenesis (Epstein et al., 2017). Inhibiting lysosomes in young testis caused the accumulation of E‐cadherin (Shg), which mimicked the natural aging process, causing abnormalities of spermatocytes (Butsch et al., 2022). A recent study also identified several factors that maintained two mitotic stem cell pools in the stem cell niche, and the cell‐cycle‐responsive DP transcription factor/E2F transcription factor 1 (Dp/E2f1) was identified as the key transcription factor for CySCs to maintain hub cell quiescence (Herrera et al., 2021). Recently, studies on testicular aging have mainly focused on gametogenesis, while few have investigated epithelial cells in TE region of *Drosophila* testis.

Hub cells, as post‐mitotic somatic cells, originate from epithelial cells, are quiescent in adult male testis, and regulate the behavior of CySCs and GSCs in *Drosophila* testis (Voog et al., 2014; White‐Cooper, 2009). Previous studies also indicated that hub cells exited quiescence and transformed into CySC‐like cells after the ablation of CySCs (Hétié et al., 2023). Several research groups discovered a common pattern from scRNA‐seq data in the *Drosophila* testis. Notably, several markers, e.g. Fasciclin III (Fas3), Shg, and hedgehog (hh), share similar expression patterns in hub cells and epithelial cell clusters (Raz et al., 2023; Witt et al., 2019; Yu, Fu, et al., 2023; Yu, Li, et al., 2023). Importantly, hh, Fas3, and Shg are highly enriched in hub cells and are required for stem cell maintenance in the niche (Lee et al., 2016; Michel et al., 2012; Voog et al., 2008). Moreover, these proteins are also associated with cell–cell adhesion and junction complexes in hub cells (Boyle et al., 2007; Le Bras & Van Doren, 2006; Papagiannouli & Mechler, 2009). Notably, Shg acts as the main component of adherens junctions and helps to tightly link epithelial cells together via the extracellular matrix (ECM) (Cabrera et al., 2023; Harris & Tepass, 2010; Mendonsa et al., 2018). These data indicated that hub cells and epithelial cells might originate from the same type of cell ancestors.

Septate junctions are evolutionary conserved, with striking similarities in their molecular constituents among species (Banerjee et al., 2006). Transferrin 2 (Tsf2) is a phylogenetically conserved iron‐binding protein that attaches to epithelial cell membranes, and is essential for the assembly of paracellular septate junctions (Tiklová et al., 2010). In *Drosophila* testis, during the elongated spermatid stage, septate junctions could be observed between somatic cyst cells (Dubey et al., 2019). Septate junction‐related proteins localize at the head and tail of cyst cells at the base of the testis to support the mechanical stability of the somatic enclosure and prevent abnormal spermatid release (Dubey et al., 2019).

The regulatory mechanisms of the TE region, which is the key epithelial cell cluster at the base of the testis, remain largely undiscovered. Therefore, we identified TE populations by their *MtnA* expression, which was verified to show a specific expression pattern in the TE region (Raz et al., 2023). *MtnA* encodes a metallothionein, the expression of which is strongly stimulated by copper and cadmium ions, and whose induction depends on the binding of Metal response element‐binding Transcription Factor‐1 (MTF‐1) to the *MtnA* promoter (Sims et al., 2012). The scRNA‐seq analysis showed that TE populations had multiple functions, including support, nutrition, and antioxidant activity. Thus, epithelial cells in the TE region might self‐regulate via multiple signaling pathways, such as the Ras signaling pathway, Rap1 signaling pathway, and Toll and Imd signaling pathway. Specifically, the EP0 population comprised the highest number of epithelial cells in the TE region, and was predicted to have key roles related to tight junctions, focal adhesion, and bacterial invasion, which might constitute the primary functions for testicular TE populations in *Drosophila*. Interestingly, we also identified novel roles for the EP1, EP3, and EP5 TE populations, such as multiple metabolic processes, oxidative stress, mitochondrial function, proteasome, and apoptosis, thereby expanding our understanding of the TE region in *Drosophila* testis.

We next asked whether age‐related changes to TE cells are crucial for testicular aging in the *Drosophila*. According to the results of DEGs between 2 days and 40 days groups, we also identified diverse functions of TE during aging. These novel signatures included mitochondria‐related events, protein synthesis and degradation‐related processes, and metabolic processes. In the pseudotime trajectory analysis of testicular TE populations during aging, we noted that *Ssk* was a state‐biased marker gene in cluster 3. Ssk is essential for the formation of septate junctions, and could interact with septate‐junction‐specific component Mesh to maintain their localization in the septate junctions of epithelial cells (Izumi et al., 2012). Moreover, another component of septate junctions, Tetraspanin 2A (Tsp2A), could cooperate with Mesh and Ssk to form a complex that is essential for the organization of septate junctions (Izumi et al., 2016).

In this study, we further explored whether there is variation in the functional constraints of age‐biased genes during testicular TE aging, and used the ratio of non‐synonymous to synonymous substitutions (dN/dS) values to compare *Drosophila* divergence and selection. Gene sets with high dN/dS values usually prompt that positive selection quickly promote the fixation of their encoded amino acids when organisms better adapt to the surrounding environment (Stanley Jr. & Kulathinal, 2016). Previously, Witt et al., found that genes showing biased expression in young germ cells had higher dN/dS values than genes showing biased expression in old germ cells (Witt et al., 2023). Surprisingly, our data also demonstrated that age‐biased genes in epithelial cells of young (2 days) group evolved rapidly and were endowed with greater evolutionary advantages. This suggests that rapidly evolving genes are often expressed in young testicular TE populations and are subject to antagonistic pleiotropy duing aging with roles in gene expression shift (Austad & Hoffman, 2018).

In summary, the above evidences enhanced our understanding of the functionality of epithelial cells in the TE region of fruit fly testis. Our results represent a good resource for further research into the testicular biology of *Drosophila*.

## 4 MATERIALS AND METHODS

### 4.1 Fly stocks and crosses

*Drosophila w* ^*1118*^ flies were fed standard corn syrup medium and raised under conditions of 40–60% relative humidity, a synchronized 12:12 h light–dark cycle and room temperature. Male flies at 2, 10, and 40 days were used for further analysis.

The transgenic RNAi flies were obtained from TsingHua Fly Center (THFC). Detailed information of UAS‐RNAi and tool flies were as follows: (1) *yw; esg‐Gal4, UAS‐GFP/CyO*. (2) *UAS‐blw RNAi* (TH201500077.S). (3) *UAS‐PCB RNAi* (TH02733.N). (4) *UAS‐Fum3 RNAi* (TH05141.N). Two‐ to three‐day‐old flies were selected for mating. UAS‐Gal4 crosses were set and raised at 25°C. Male esg‐Gal4 transgenic flies were crossed with the virgin UAS‐RNAi females. Qualified male offspring with esg‐Gal4 driver and UAS‐RNAi were chosen for the further functional analysis. Male fertility test was performed by using a single *esg > RNAi* adult male that was enclosed for 3 days in a cross with three *w* ^*1118*^ virgin females at room temperature. The *esg >* males were used as the control.

### 4.2 Sample collection, tissue disruption, and preparation of a single‐cell suspension

We dissected out the testes of the 40 days group, rinsed them using cold phosphate‐buffered saline (PBS; HyClone, Logan, UT, USA), and transferred them immediately into ice‐cold GEXSCOPE™ Tissue Preservation Solution (Singleron Biotechnologies, Cheshire, CT, USA). The testes were then rinsed thrice using Hanks Balanced Salt Solution (HBSS), digested in 2 mL of GEXSCOPE™ Tissue Dissociation Solution (Singleron Biotechnologies) in a Singleron PythoN™ Automated Tissue Dissociation System for 15 min at 28°C, subjected to centrifugation for 5 min at 500 × *g*, and suspended in PBS. Trypan blue (Sigma‐Aldrich, St. Louis, MO, USA) was used to stain the samples, whose viability was determined under a microscope.

### 4.3 Preparing the scRNA sequencing library

A Singleron Matrix® Single Cell Processing System was used to load single‐cell suspensions (1 × 10^5^ cells/mL in PBS) into microfluidic devices. The protocol of the GEXSCOPE® Single Cell RNA Library Kit was then followed for scRNA‐seq library construction. Briefly, the single‐cell suspension was added into the microchip to allow partitioning the single cells into individual wells. Next, we loaded Cell barcoding beads into the microchip cells and washed them. Then, the cells were lysed using 100 μL of single‐cell lysis buffer in each well, followed by mRNA capture for 20 min at room temperature. The mRNA‐bearing beads were extracted from the microchip and subjected to reverse transcription and amplification of cDNA to construct the libraries. The cDNAs were purified, size selected, and pooled before sequencing using an Illumina Novaseq 6000 (San Diego, CA, USA) to generate 150‐bp paired‐end reads.

### 4.4 scRNA‐seq quantifications and analysis of scRNA‐seq data

Raw data of the 2 days and 10 days groups were obtained from previous studies (Yu, Fu, et al., 2023; Yu, Li, et al., 2023). An internal pipeline was used to process the raw reads of the 2, 10, and 40 Day groups to provide gene expression profiles. Briefly, reads without poly‐T tails were filtered out, and the UMI was extracted for each cell barcode. For those reads with the same cell barcode, the gene and UMI were grouped together to determine the number of UMIs per gene in each cell. Further analysis was carried out using UMI count tables for each cellular barcode. We filtered out those cells with a high mitochondrial gene percent (> 25%) or an unusually large number of UMIs (> 60,000). Cells containing fewer than 510 or more than 6000 genes were also excluded.

The Seurat program (Butler et al., 2018; Satija et al., 2015) was then employed for clustering analysis and cell type identification. For each sample, we imported individual cell‐by‐gene matrices for each sample into Seurat version 3.1.1 for downstream analysis (Butler et al., 2018). Cell clusters were visualized using t‐distributed Stochastic Neighbor Embedding (t‐SNE). Significantly upregulated enriched genes were identified using criteria of fold change >1.28 and *p*‐value <0.01. A fold change >2 and a P‐value <0.05 identified significantly DEGs. clusterProfiler software was used for Gene Ontology (GO) and Kyoto Encyclopedia of Genes and Genomes (KEGG) analyses of the DEGs to explore their biological functions or significant pathways (Yu et al., 2012). Monocle 2 (version 2.10.1) was employed for single cell trajectory analysis based on gene expression and the matrix of the cells, to reduce the research space to one with two dimensions and to order the cells (Qiu et al., 2017; Trapnell et al., 2014). To identify key factors or pathways, cell differentiation trajectories were assessed using pseudotime trajectory analysis.

### 4.5 RNA extraction and quantitative real‐time reverse transcription PCR (qRT‐PCR)

Briefly, the TRIzol reagent (15,596,026; Invitrogen, Waltham, MA, USA) was used to extract total RNA from testes. A PrimeScript™ II 1st Strand cDNA Synthesis Kit (6210A; Takara, Shiga, Japan) was then used to synthesize cDNA from the total RNA. The qPCR step of the qRT‐PCR protocol was carried out using a LightCycler® 96 Real‐Time PCR System (Roche, Basel, Switzerland) with TB Green Premix Ex Taq II (RR820; Takara). The primer sequences used are shown in Table S4.

### 4.6 Immunostaining

Fly testes were dissected in 1 × phosphate‐buffered saline (PBS), and fixed for 30 min in 4% paraformaldehyde, washed with 0.3% PBS‐Triton X‐100 (PBST) three times, and blocked in 5% bovine serum albumin (BSA) for 30 min. Testes were incubated with mouse anti‐FasIII (Developmental Studies Hybridoma Bank, 1:50) antibody at room temperature for 1 h. Then, testes were washed with three times in 0.3% PBST and incubated with secondary antibody conjugated with A647 (Jackson ImmunoResearch Laboratories, 1:800) at room temperature for 1 h in the dark. After washing three times again with 0.3% PBST, testes were stained with Hoechst‐33,342 (Solarbio, 1.0 mg/mL) for 5 min before mounting.

### 4.7 Fluorescence in situ hybridization (FISH)

FISH probe mix was synthesized by Ribo Bio Technology Co Ltd (Guangzhou, China). FISH was performed with the FISH kit according to the manufacturer's protocol. Testes were fixed with 4% paraformaldehyde for 30 min at room temperature, and then permeabilized in PBS with 1% Triton X‐100 on ice for 30 min. Followed by pretreatment with pre‐hybridization buffer at 37°C for 30 min. Subsequently, testes were hybridized with 20 μM using Cy3‐labeled RNA of FISH probe mix in a moist chamber at 37°C overnight. Testes were rinsed thrice in 4 × SSC with 0.1% Tween‐20 for 5 min at 42°C, washed once for 5 min at 42°C in 2 × SSC and then washed once for 5 min at 42°C in 1 × SSC. After hybridization, testes were stained with Hoechst‐33,342 (Solarbio, 1.0 mg/mL) for 5 min before mounting.

### 4.8 Statistical considerations

Quantitative data are shown as the mean ± standard error of mean (SEM) in GraphPad Prism software version 6.01 (GraphPad Inc., La Jolla, CA, USA). One‐way analysis of variance (ANOVA) was conducted to examine comparisons for more than two groups using Dunnett's multiple comparison test. In the figures, one asterisk indicates *p* < 0.05; two asterisks indicate *p* < 0.01; and three asterisks indicate *p* < 0.001.

## AUTHOR CONTRIBUTIONS

Xia Chen, Jun Yu, Yijuan Cao, and Zhifeng Gu initiated the project, designed the study, coordinated the experiment, and wrote the manuscript. Yujuan Qi, Qiuru Huang, Chi Sun, Yanli Zheng, Li JiL., Yi Shi, and Xinmeng Cheng performed the experiments and provided conceptual inputs for the paper. Jun Yu Xia Chen, Zhenbei Li, and Sen Zheng. analyzed the data. All authors read and approved the final manuscript.

## FUNDING INFORMATION

This study was funded by Natural Science Foundation of Jiangsu Province (BK20221376), National Natural Science Foundation of China (82071838, 82101891), Jiangsu province senile health scientific research project (LR2021035), Basic Science Research Program of Nantong City (JC12022006), Science and technology Project of Nantong City (JC2021119), Research Project of Nantong Health Commission (QA2021016), the Fund of Xuzhou Science and Technology (KC20092), Workstation of Academician Liu Yixun (2–2018.11.1), Xuzhou Pengcheng Talents‐Medical Youth Reserve Talent Project (XWRCSL20220162), the Nantong Key Young Medical Talent Program, the Open Fund of Jiangsu Key Laboratory for New Drug Research and Clinical Pharmacy.

## CONFLICT OF INTEREST STATEMENT

The authors declare that they have no known competing financial interests or personal relationships that could have appeared to influence the work reported in this paper.

## Supporting information


**Data S1.** Supporting Information

**Table S1.** The nUMIs of sub‐cell clusters.

**Table S2.** The nGenes of sub‐cell clusters.

**Table S3.** The average expressions of key marker genes for Dotplot view.

**Table S4.** Detailed primers used for qRT‐PCR.

## Data Availability

The data that support the findings of the current study are available from the corresponding author upon reasonable request.
