# Supplementary material for: Single‐cell transcriptome characteristics of testicular terminal epithelium lineages during aging in the Drosophila
Source: Aging Cell. 2023 Dec 3;23(3):e14057. doi: 10.1111/acel.14057 (PMC10928582; doi:10.1111/acel.14057)
Supplement: Supplementary file 1 — Data S1. Supporting Information [file ACEL-23-e14057-s002.pdf]

Supplementary materials

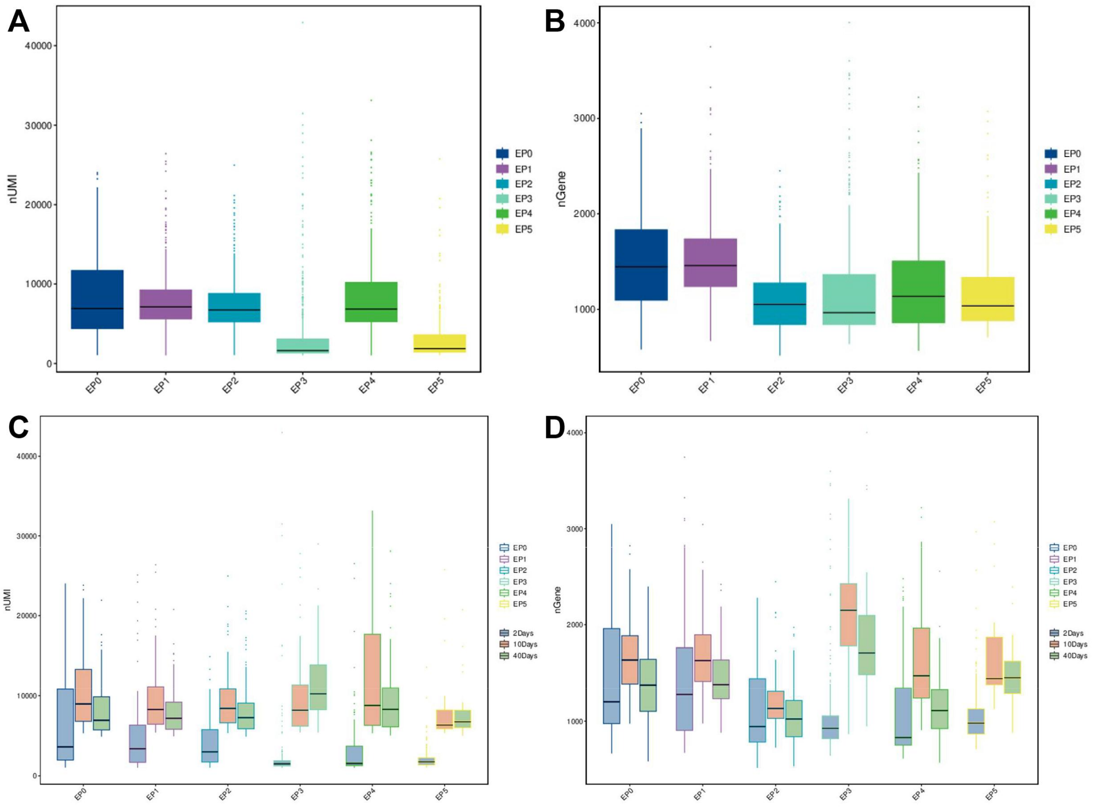

Figure S1. Quality control of testicular TE populations. (A) RNA contents for each sub-cluster of testicular TE populations. (B) Gene expression levels for each sub-cell cluster of testicular TE populations. (C) RNA contents for testicular TE populations during aging. (D) Gene expression levels for testicular TE populations during aging.

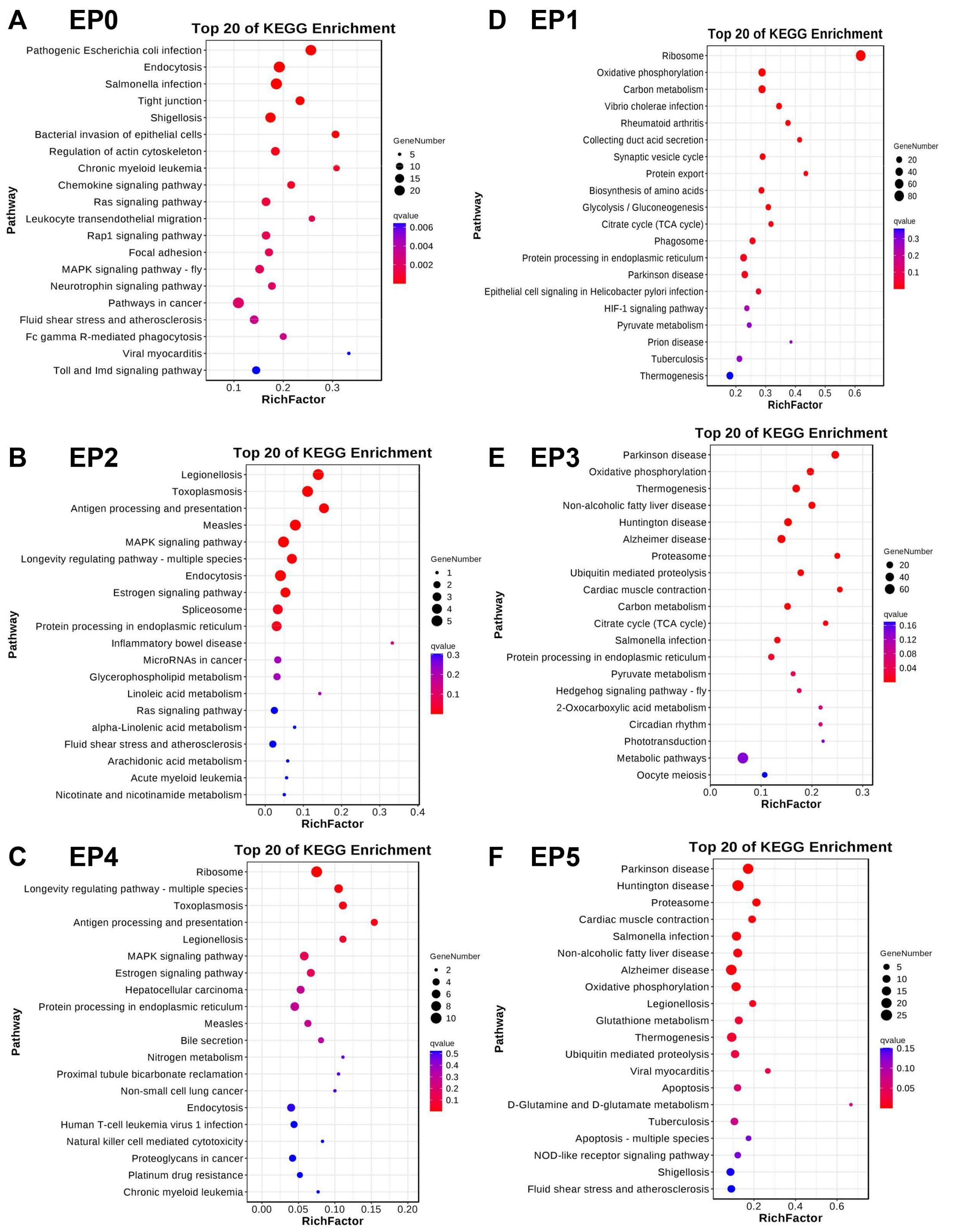

Figure S2. Enrichment analysis of marker gene collections. Top 20 enriched KEGG pathways in EP0 (A), EP2 (B), EP4 (C), EP1 (D), EP3 (E), and EP5 (F) TE populations.

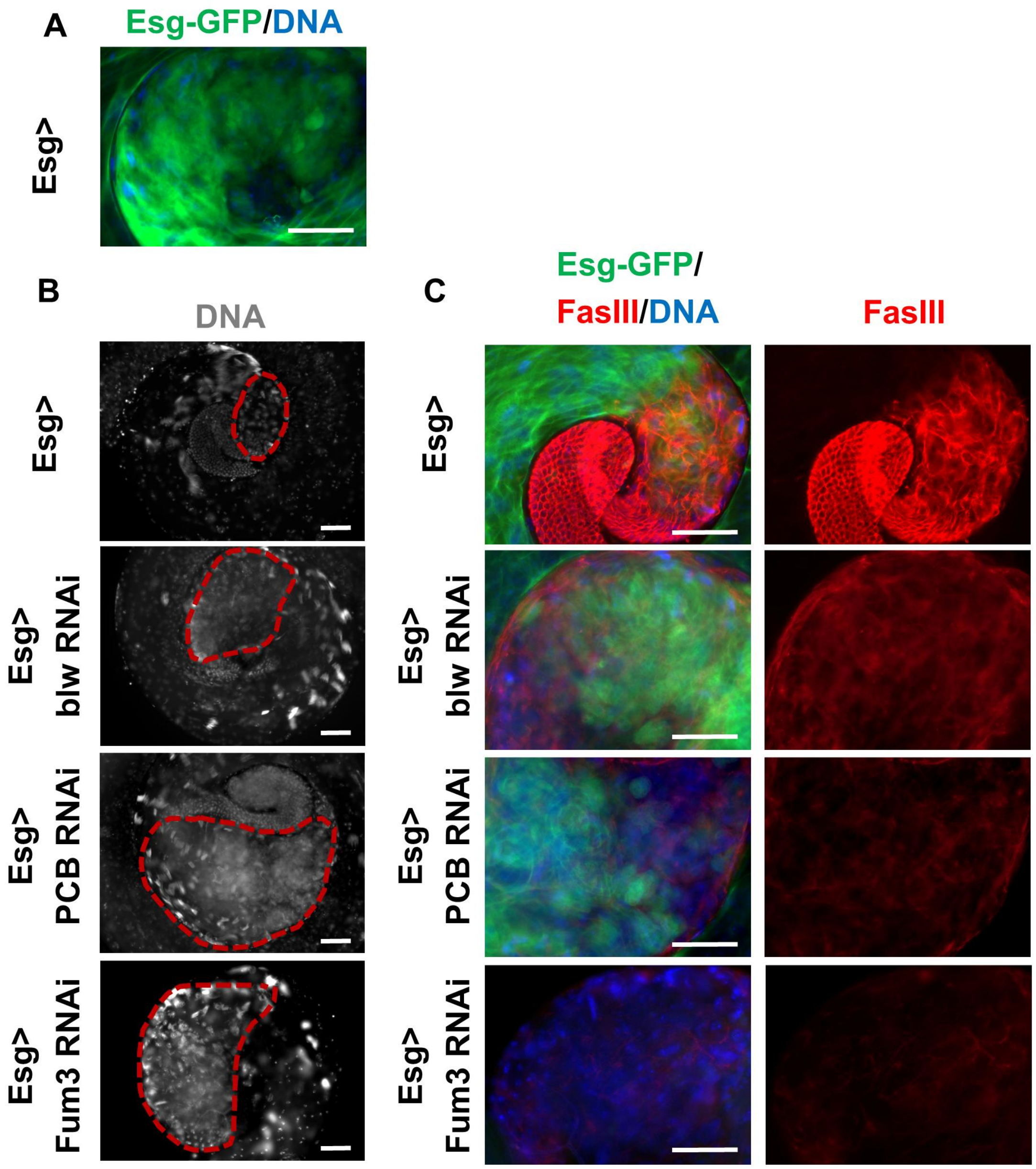

Figure S3. Phenotypic analysis of knocking down candidate genes of pathways involved. (A) Esg-GFP expression pattern in TE populations of esg>GFP testis. (B) DNA staining of TE regions in *esg>*, *esg>blw RNAi*, *esg>PCB RNAi*, and *esg>Fum3 RNAi* testes. The red circles represent the TE regions. (C) Immunostaining of Esg-GFP and FasIII in TE regions of *esg>*, *esg>blw RNAi*, *esg>PCB RNAi*, and *esg>Fum3 RNAi* testes. Scale bars: 50 μm.

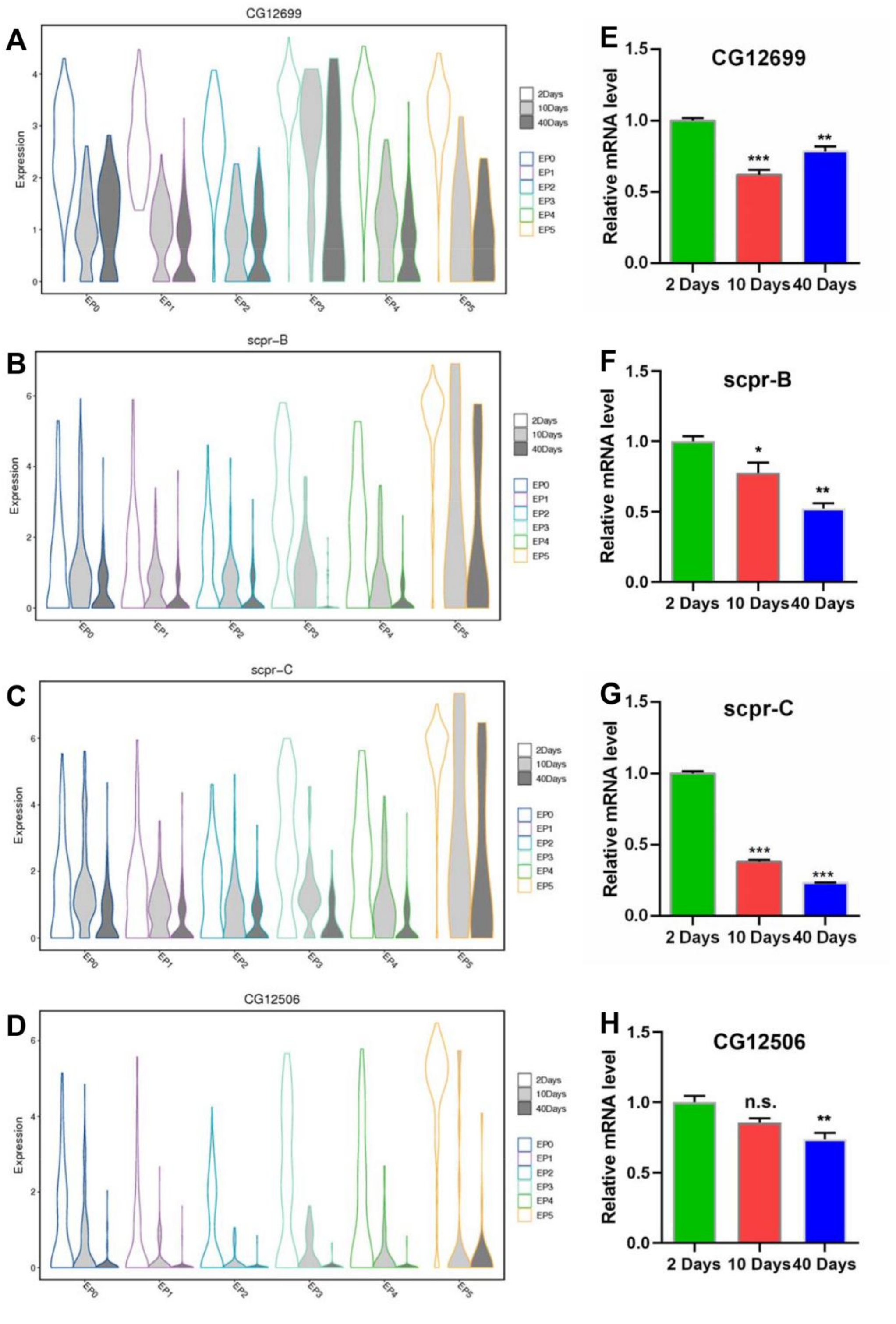

Figure S4. Analysis of expression patterns of selected genes during aging. (A-D) Violin plots of *CG12699* (A), *Scpr-B* (B), *Scpr-C* (C), and *CG12506* (D) for each sub-cell cluster of testicular TE populations during aging. (E-H) Relative mRNA levels of *CG12699* (E), *Scpr-B* (F), *Scpr-C* (G), and *CG12506* (H) in *Drosophila* testes. *$P < 0.05$, **$P < 0.01$, ***$P < 0.001$.

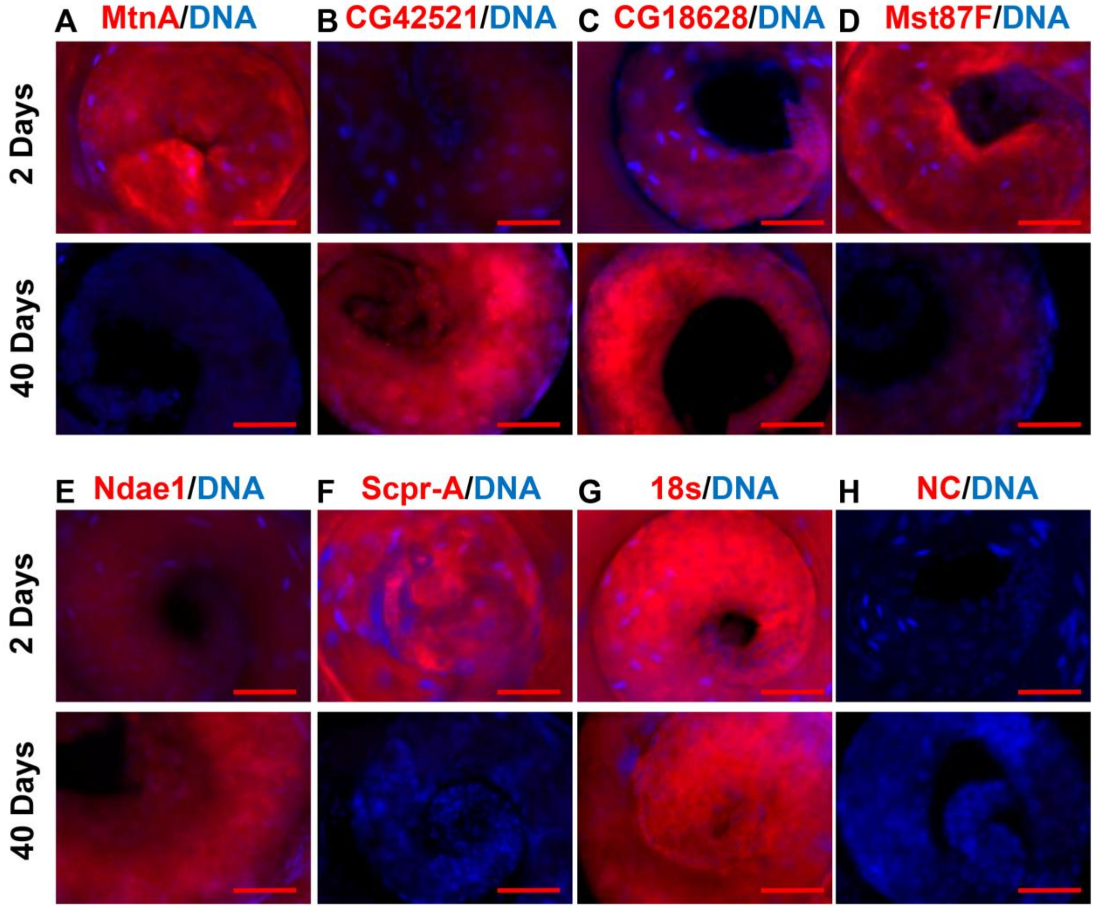

Figure S5. Expression patterns of representative marker genes in testicular TE regions during aging. FISH images showing localizations and expression patterns of *MtnA* (A), *CG4252*1 (B), *CG18628* (C), *Mst87F* (D), *Ndae1* (E), *Scpr-A* (F), *18s* (G), and NC (H) probes in testicular TE regions in the 2 Days and the 40 Days groups. *18s*, probe for *18S rRNA*; NC probe was used as negative control. Scale bars: 50 μm.

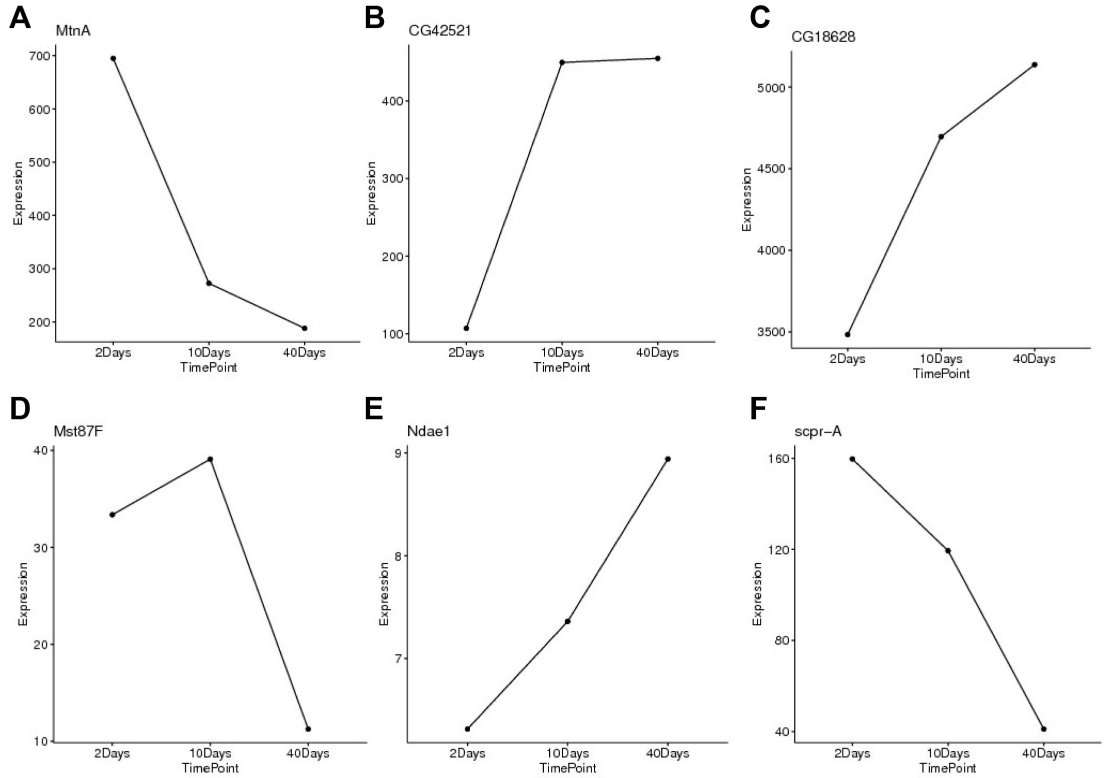

Figure S6. Expression trends of representative marker genes. Expression levels of *MtnA* (A), *CG42521* (B), *CG18628* (C), *Mst87F* (D), *Ndae1* (E) and *Scpr-A* (F) in testicular TE populations at 2, 10 , and 40 Days.

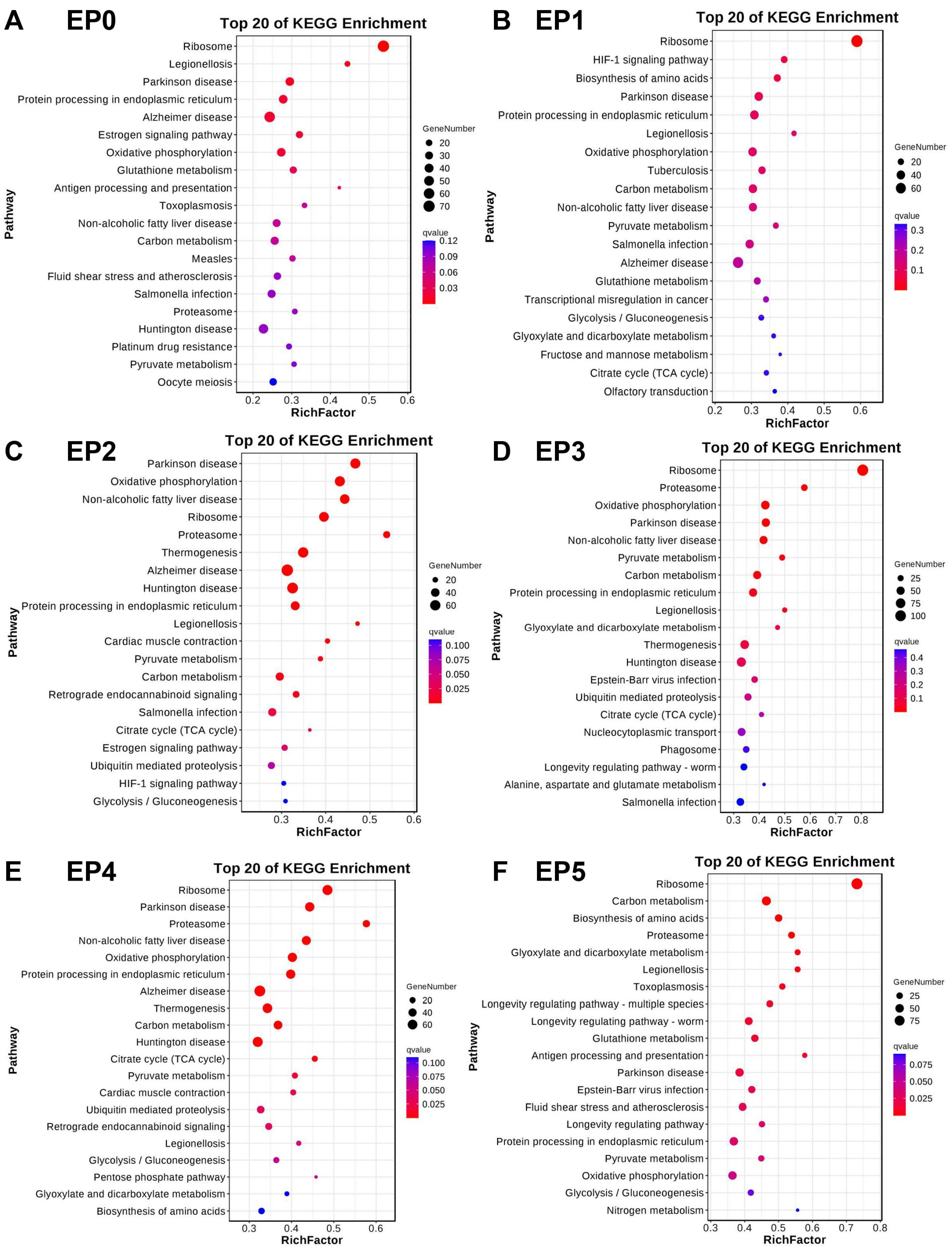

Figure S7. Enrichment analysis of DEGs for testicular TE populations during aging. Top 20 enriched KEGG pathways for EP0 (A), EP1 (B), EP2 (C), EP3 (D), EP4 (E), and EP5 (F) TE populations between the 2 Days and 40 Days groups in *Drosophila*.

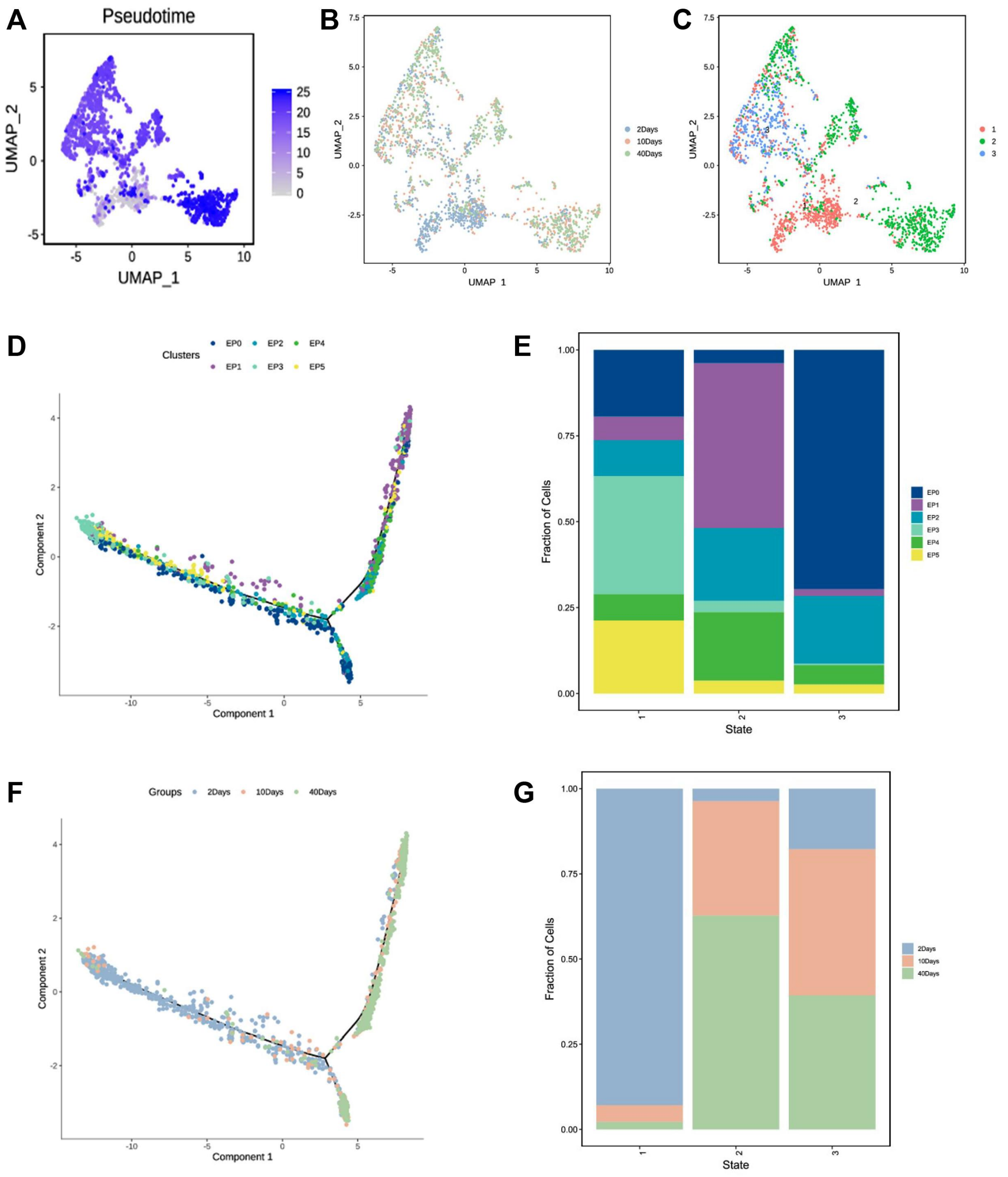

Figure S8. Pseudotime trajectory analysis of testicular TE populations. (A-C) Pseudotime trajectory analysis visualized using UMAP based on pseudotime (A), group (B) and cluster (C). (D) Testicular TE populations pseudotime trajectory analysis colored by the various sub-populations. (E) Cellular components of testicular TE sub-populations in each cluster. (F) Testicular TE populations pseudotime trajectory analysis colored by the various groups. (G) Cellular components of testicular TE populations by different groups in each cluster.

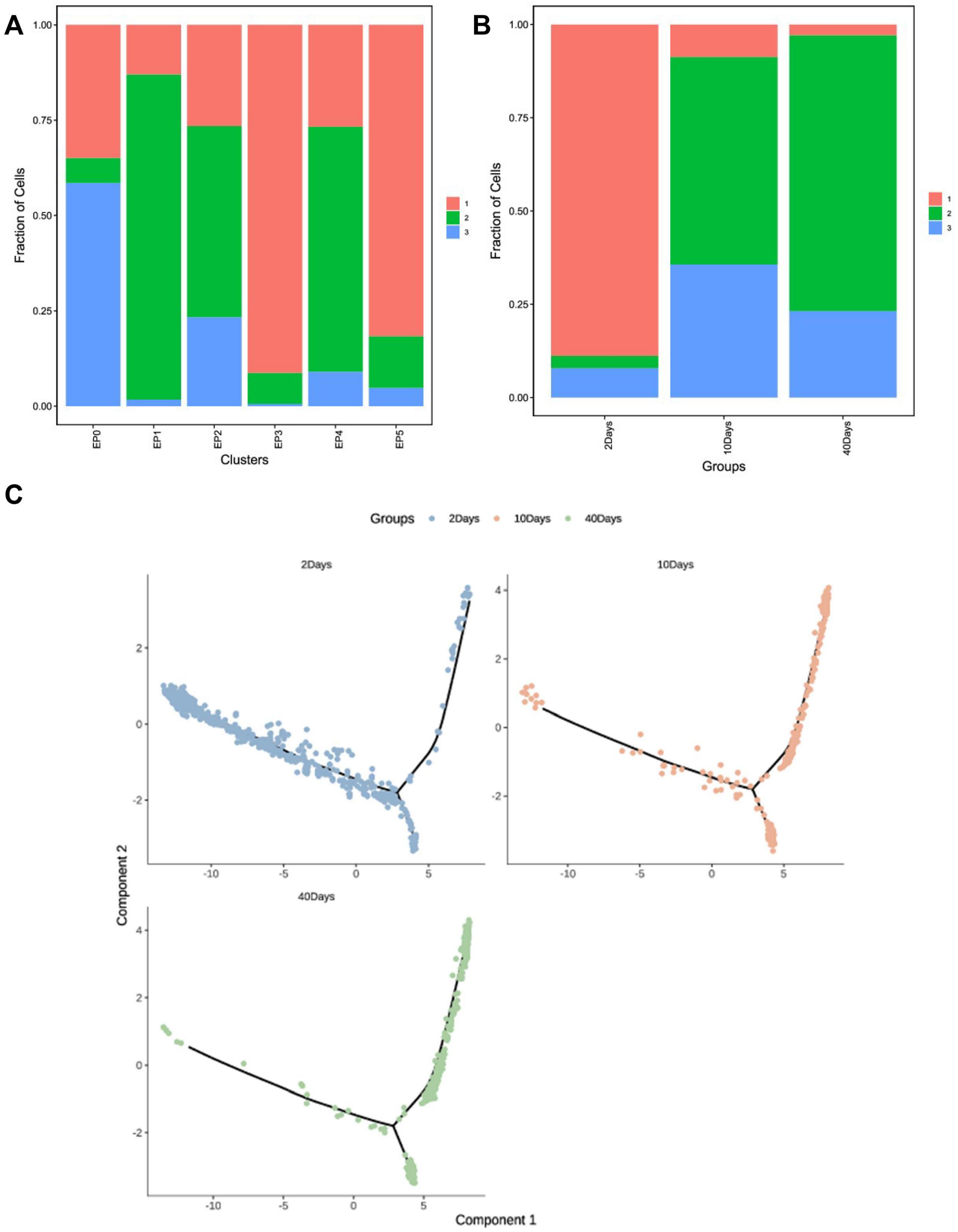

Fig. S9. Cellular component analysis for the three clusters of testicular TE populations. (A) Cellular components of the three clusters in each TE sub-population. (B) Cellular components of the three clusters in each group. (C) The single-cell trajectory analysis of testicular TE populations in each group. The distribution of each group is shown separately.

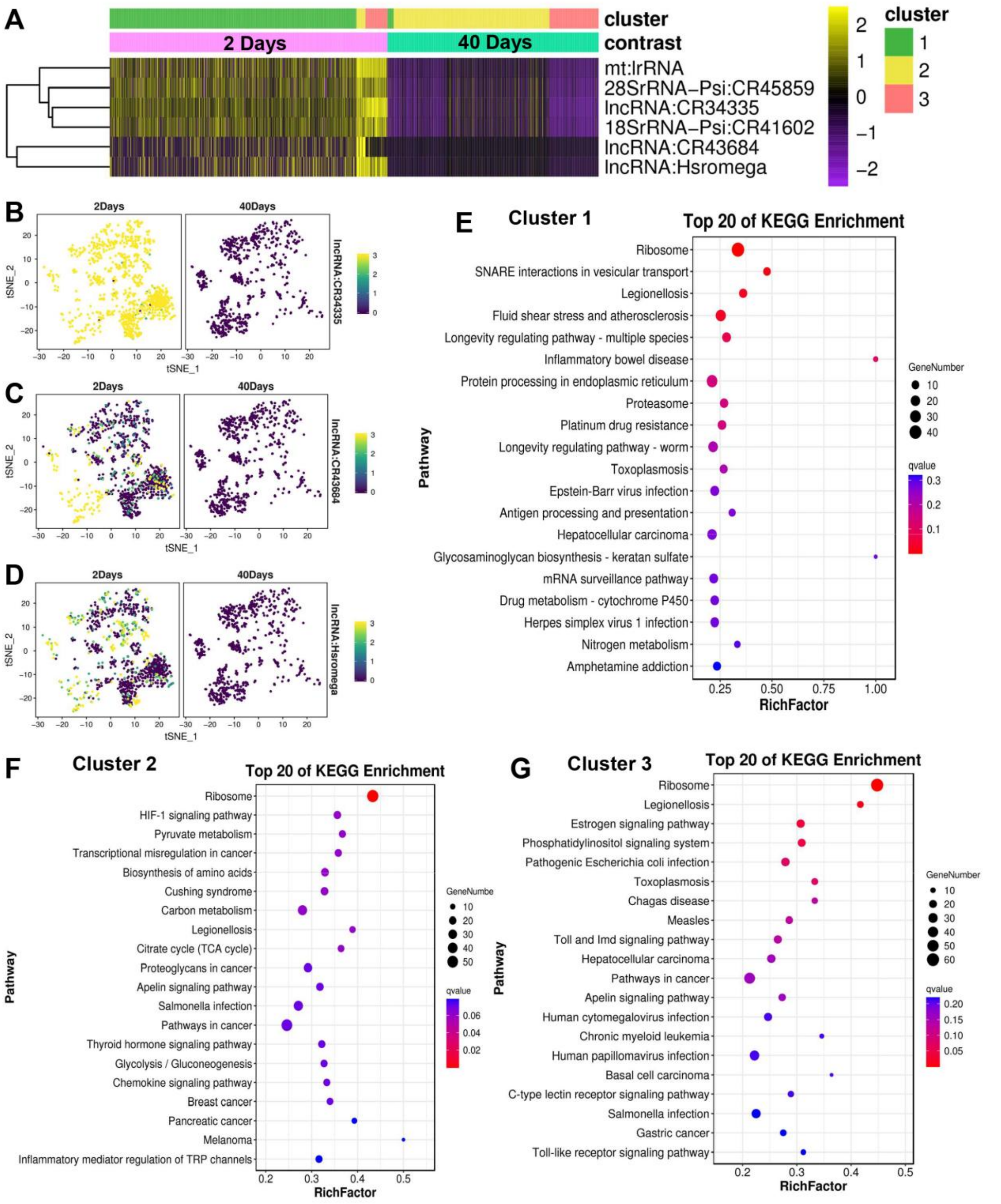

Figure S10. DEGs analysis of testicular TE populations during aging. (A) Heatmap view of representative enriched DEGs between the 2 Days and 40 Days groups for testicular TE populations in the three clusters. (B-D) tSNE visualizations of *lncRNA:CR34335* (B), *lncRNA:CR43684* (C) and *lncRNA:Hsromega* (D) genes in the 2 Days and 40 Days groups. (E) Top 20 enriched KEGG pathways for DEGs in cluster 1 TE populations. (F) Top 20 enriched KEGG pathways for DEGs in cluster 2 TE populations. (G) Top 20 enriched KEGG pathways for DEGs in cluster 3 TE populations.

Table S1.The nUMIs of sub-cell clusters.

Table S2.The nGenes of sub-cell clusters.

Table S3. The average expressions of key marker genes for Dotplot view.

Table S4. Detailed primers used for qRT-PCR.
