# Supplementary material for: Single‐cell transcriptome characteristics of testicular terminal epithelium lineages during aging in the Drosophila
Source: Aging Cell. 2023 Dec 3;23(3):e14057. doi: 10.1111/acel.14057 (PMC10928582; doi:10.1111/acel.14057)
Supplement: Supplementary file 5 — Table S4. Detailed primers used for qRT‐PCR. [file ACEL-23-e14057-s001.doc]

**Table S4. Detailed primers used for qRT-PCR.**

| **Gene** | **Forward (5'-3')** | **Reverse (5'-3')** |
| --- | --- | --- |
| MtnA | TCGTGGAAATGGGATGGTCAAGTG | CAGTCGCTTCAGAAGTGAGGCATC |
| CG42521 | TAGACCTGGCAGCGGAAGACC | TGCTCCTCCATCACCTCCACTTC |
| CG18628 | CAAGGCATCAGAAGCAAGCAAGC | TTCAGTTGGGGAAGGGCAGAGG |
| Mst87F | CCTGTGGCGGCTGCTATTGTG | GGTGTTCTGGTGAACCCTTCTTGG |
| Ndael | TTGGTTGGTGGAGATTGCGGAAC | AGCACGGTGGAGATGAGGAAGG |
| scpr-A | ACTTCGTACTCACCTGCAACTTCG | CTCCTTGGCGTAGCACAGATTGG |
| scpr-B | AGCCGAGACAGAGTACACCGATG | AACTTGGTCACCGCCTTGTTGG |
| scpr-C | CAACCTGTGCTACGCCAAGGAG | GTCCATTCCATGCCTACATCGTCTG |
| CG12699 | CGGGTGAGGCTGTTGAAGAAGAAG | ATGACCTTGGTGGCATACATGGC |
| CG12506 | CTGCTCAAGGCGGACCACAAG | TCCCTGCTGACCTCCCTGTTG |
| Gapdh | GTGGTGAACGGCCAGAAGAT | GCCTTGTCAATGGTGGTGAA |
